# Spontaneous Retroperitoneal Hemorrhage Secondary to Chronic Celiac Axis Compression Treated with Embolization Utilizing Cone Beam CT

**DOI:** 10.1155/2020/2636495

**Published:** 2020-08-01

**Authors:** Bashar Khiatah, Sam Jazayeri, Charles M. Hubeny, Brian Nadav, Amanda Frugoli

**Affiliations:** ^1^Internal Medicine Department, Community Memorial Hospital Ventura, CA, USA; ^2^Interventional Radiology, Pueblo Radiology, Santa Barbara, CA, USA; ^3^Internal Medicine Department, GME, Community Memorial Hospital Ventura, CA, USA

## Abstract

Median arcuate ligament syndrome (MALS) is a rare and often misdiagnosed vascular pathology. In this paper, we discuss a 51-year-old female with MALS presenting with hypotension due to retroperitoneal hemorrhage. Currently, there is no consensus regarding the optimal treatment approach for such patients. This case report demonstrates the utility of conventional mesenteric angiography, cone beam CT with 3D reconstruction, and selective mesenteric transarterial embolization as an effective treatment approach for patients with spontaneous aneurysm rupture in MALS.

## 1. Introduction

Median arcuate ligament syndrome (MALS), also known as celiac artery compression syndrome, celiac axis syndrome, or Dunbar syndrome, is a rare and often misdiagnosed vascular pathology. The syndrome is more frequent in females in their 4th to 6th decades of life with chronic symptoms lasting from a couple of months to many years. The classic triad consists of postprandial abdominal pain, weight loss, and epigastric bruit. However, this common presentation is not always present [1, 2]. MALS can be asymptomatic in 10-24% of the patients due to a robust collateral circulation from the superior mesenteric artery (SMA) [3]. Rarely, aneurysms of the pancreaticoduodenal arteries can occur, and the initial presentation may be sudden abdominal pain, anemia, and hypotension secondary to spontaneous retroperitoneal hemorrhage. Treatment guidelines in this situation are unclear, but given the complexity of the pathology, a multidisciplinary approach is often best. Transarterial embolization can be difficult given the complex collateral anatomy but has proved to be an effective initial treatment in the acute setting. This case illustrates a rare case of spontaneous retroperitoneal hemorrhage as the first presentation of MALS successfully treated with endovascular embolization aided by cone beam CT.

## 2. Clinical Case

A 51-year-old healthy female without significant past medical history presented to the emergency department with severe nonradiating abdominal pain that began without an inciting event. The pain was mainly located in the epigastric region. The pain was described as sharp and was associated with nausea; multiple episodes of nonbloody, nonbilious emesis; chills; and a near syncopal episode. There were no specific alleviating or exacerbating factors, and she never had similar symptoms in the past. She denied any fever, dysuria, history of heartburn, peptic/duodenal ulcer disease, heavy nonsteroidal anti-inflammatory drugs (NSAID) use, recent illnesses, recent ill contacts, or recent travel. She denied any significant surgical history. She is a former heavy smoker and reported current moderate alcohol use and a recent increase in her stress level. The patient denied a prior history of EGD or colonoscopy. Family history is significant for coronary artery disease in both parents.

In the emergency department, she was found to be hypotensive with a blood pressure of 64/48 mmHg and a heart rate of 71 bpm. Her oxygen saturation was 100% on room air. Physical exam revealed a diffusely tender abdomen. Lab tests were significant for a hemoglobin of 9.1 g/dL and a white blood count of 14.4 k/*μ*L. Subsequently, 2 large-bore intravenous (IV) catheters were inserted and a blood type and screen were ordered. IV fluids were started along with pain and nausea control medications.

**Figure 1:**
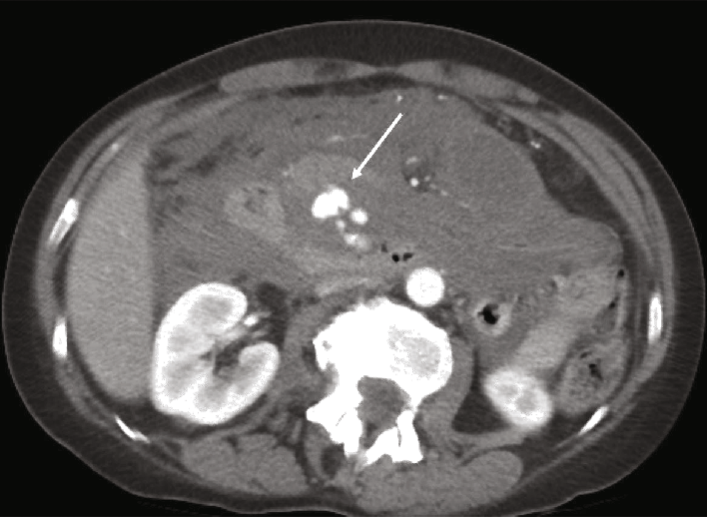
Contrast-enhanced CT shows a large retroperitoneal hemorrhage with an area of active extravasation between the duodenum and the pancreas (arrow).

**Figure 2:**
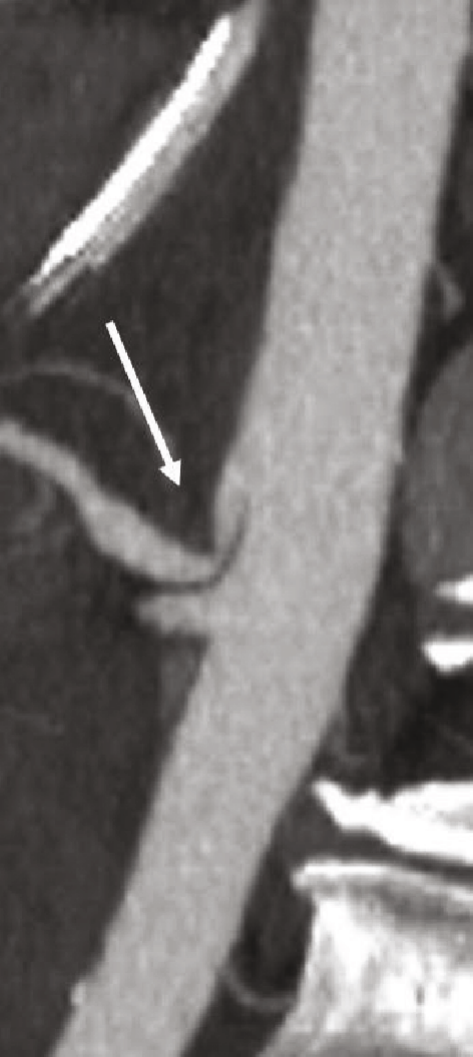
External compression of the proximal celiac artery by the median arcuate ligament is confirmed on sagittal maximal intensity imaging.

A contrast-enhanced CT scan of the abdomen and pelvis revealed a large retroperitoneal hematoma with a lobulated focus of active contrast extravasation interposed between the pancreatic head and the duodenum (Figure 1). The bleeding was localized to the retroperitoneum itself and did not involve nearby retroperitoneal organs such as the pancreas, kidneys, or duodenum; however, these organs did show displacement by the hematoma. Several nearby vessels were identified as the potential source of bleeding including the distal gastroduodenal artery as well as several prominent superior and inferior pancreaticoduodenal (PDA) arteries extending from the gastroduodenal and superior mesenteric arteries, respectively. Finally, a high-grade proximal celiac artery stenosis was noted consistent with median arcuate ligament compression (Figure 2). Interventional radiology (IR) and general surgery were consulted, and a mesenteric angiogram was emergently performed to localize the bleeding for potential embolization.

**Figure 3:**
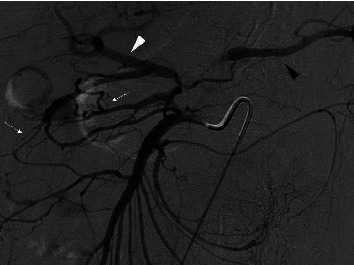
Arteriogram of the SMA demonstrates the brisk collateral flow to celiac branches including the hepatic artery proper (white arrowhead) and splenic artery (black arrowhead) via multiple pancreaticoduodenal collaterals (small white arrows).

**Figure 4:**
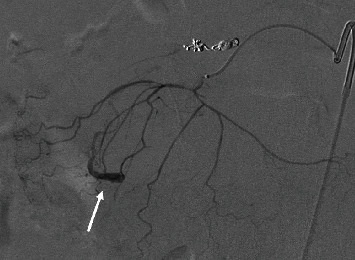
A small aneurysm was discovered with the interrogation of a distal branch of the inferior PDA.

Mesenteric angiography confirmed median arcuate ligament compression that was well compensated through brisk extensive collateral flow from the SMA via the pancreaticoduodenal arcade (Figure 3). Embolization of an inferior pancreaticoduodenal (PDA) branch was performed with detachable 0.018-inch coils given active extravasation. Further interrogation showed the abnormal aneurysmal pancreaticoduodenal vessel, but the structure was unable to be reached given only a small network of feeding arteries measuring 1 mm or less (Figure 4). No further bleeding was identified. Although the patient's condition immediately improved following embolization, there was still potential for further hemorrhage given the untreated aneurysm. Since this, after hours, treatment was prolonged with both resuscitation and procedure time, and the patient improved clinically; she was transferred to the ICU for observation and further workup. An arterial sheath was left in place for blood pressure monitoring and potential subsequent access given an incomplete interrogation of the complex anatomy. Initial management included intravenous proton pump inhibitor, placement of the nasogastric tube, and order for the serial blood counts, and a gastroenterology consult was obtained.

Later that day, the patient complained of continued epigastric abdominal pain and nausea, but vital signs remained stable. A follow-up CT scan with contrast was ordered and showed the hematoma was slightly larger containing a new bilobed 2 cm saccular area of circulating contrast. This was defined as a residual saccular pseudoaneurysm, rather than a true aneurysm, given new morphology when compared to the prior scan, and likely contained by the hematoma within the tight retroperitoneal tissues. The nearby fusiform aneurysm was not seen, presumably decompressed from rupture. The patient also required a blood transfusion given slowly dropping hemoglobin values. Although vital signs were stable, angiography was again performed given her laboratory deterioration and CT findings. The new pseudoaneurysm was identified, but unfortunately the feeding vessel remained occult after integration of multiple branches including several branches of the inferior pancreaticoduodenal artery. Given the procedure time, contrast dose, and continued relative stability of the patient, the procedure was terminated.

**Figure 5:**
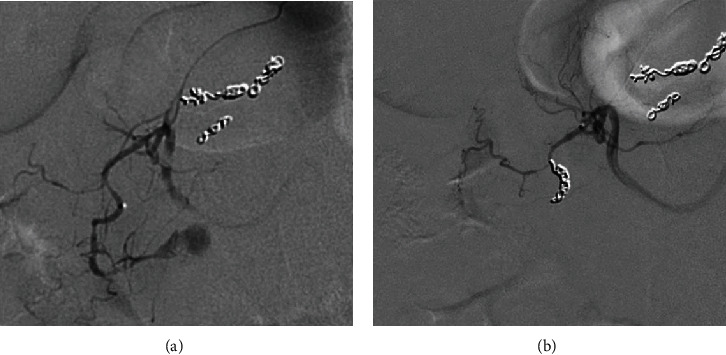
(a, b) Bilobed saccular pseudoaneurysm (arrow) was seen with the interrogation of the superior PDA, and this feeding vessel was subsequently embolized.

**Figure 6:**
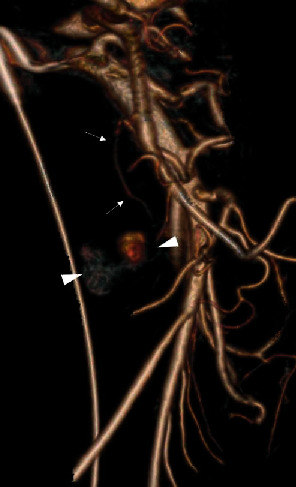
3D volume rendering of SMA arteriogram reveals a DSA occult small accessory inferior pancreaticoduodenal artery feeding the bilobed pseudoaneurysm.

**Figure 7:**
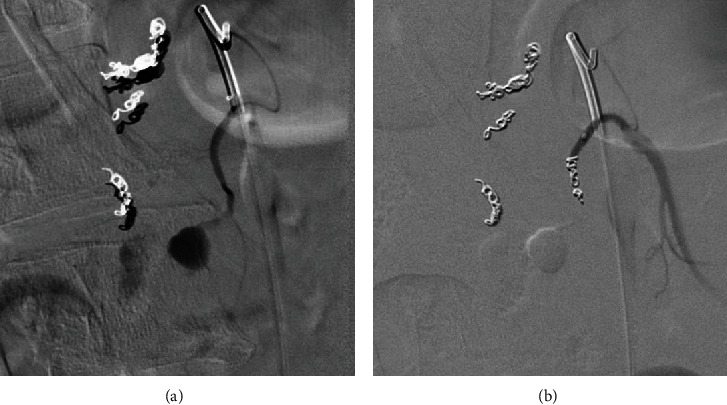
(a, b) Pre- and postembolization of the final inferior PDA feeding the bilobed PSA (arrowheads) which finally led to complete thrombosis.

The vascular surgery team decided against celiac release in the acute setting given the risks of the surgery and current blood loss but agreed that a median arcuate ligament release would likely be needed as an outpatient. The patient remained stable, but there was continued suspicion for a slow hemorrhage from the ruptured aneurysm. A third mesenteric angiogram was again performed on day 2 but this time with angiography of the celiac artery and gastroduodenal artery (GDA) via the high-grade celiac artery stenosis. Using digital subtraction angiography (DSA), a prominent superior PDA branch supplying the aneurysm was identified and treated with coil embolization (Figures 5(a) and 5(b)). Access from the celiac was removed, and SMA angiography demonstrated persistence of the aneurysm. Given the complexity of the PDA anatomy, cone beam CT angiography (GE Innova 540) with subsequent 3D reconstruction was performed. The 2D maximum intensity projection (MIP) images not only clearly demonstrated the PSA but also showed a second culprit posterior inferior PDA supplying the pseudoaneurysm. The gantry angle was optimized from the 3D volume rendering (Figure 6), and image overlay of live fluoroscopy allowed for quick vessel selection with the microcatheter/microwire. After verification with DSA, the vessel was treated with coil embolization (Figures 7(a) and 7(b)) thrombosing the PSA.

The following day, she reported feeling well with less pain and nausea. Her hemoglobin and vital signs were stable. NG was removed, and her diet was advanced. The remainder of her hospital stay was unremarkable other than hypertension treated with lisinopril, hydrochlorothiazide, and metoprolol to aid in avoiding further rebleeding events.

## 3. Discussion

The median arcuate ligament (MAL) is a ligament formed by tendinous fibers between the right and left diaphragmatic crura, which also form the ventral arch of the aortic hiatus of which four variants have been reported. An abnormal fibrous thickening of the MAL can extrinsically compress 70 to 100% of the celiac trunk lumen which has been previously reported in necropsy findings and surgical descriptions [4]. This variant anatomy is a common incidental CT finding. In MALS, the celiac origin is severely compressed by the median arcuate ligament reducing blood supply in the celiac territory. Subsequently, the intraluminal pressure difference between the SMA and the celiac arteries results in increasing compensatory blood flow across the pancreaticoduodenal arcade [5]. This pathophysiology is thought to induce the increase in arterial blood pressure and secondarily weaken the arterial walls resulting in aneurysmal dilation [6].

Many modalities have been used to diagnose MALS including mesenteric ultrasound, computed tomography angiography (CTA), magnetic resonance angiography (MRA), and conventional angiography. CTA and conventional angiography have evolved into the gold standard in the emergent setting for the diagnosis of MALS with hemorrhage. Conventional angiography also carries the advantage of treating the bleeding source. In addition, there is growing use of intraprocedural cone beam CT with 3D applications during most vascular procedures including treatment of intracranial aneurysms, allowing precise tumor treatment during hepatic chemoembolization and radioembolization [7] and defining the complex and variable anatomy during prostate artery embolization [8].

Currently, there is no consensus on the standard treatment approach for spontaneous aneurysm rupture in MALS with some authors proposing observation [9], surgical aneurysm ligation/resection, acutely relieving the celiac stenosis [10], endovascular embolization of the aneurysm alone, or embolization followed by ligament release. Percutaneous transluminal angioplasty with stent implantation has been described for treatment in the acute setting to aid with embolization as well as for chronic symptoms [11] but is rarely used for fear of fatigue and fracture due to diaphragmatic motion. Open laparotomy or video laparoscopic surgical procedures remain the most common treatment in those with chronic symptoms [12]. While MALS patients might benefit more from surgical treatments based on direct visualization and division of the arcuate ligament to achieve decompression of the celiac artery, the treatment of choice will always be individualized based on the patient's presentation [13]. Ruptured PDA aneurysm secondary to MAL is extremely rare, and classic treatments included surgical aneurysmectomy, with or without reconstruction. However, rapid advances in interventional radiology have enabled the safe and effective treatment of visceral aneurysms and acute hemorrhage via transcatheter arterial embolization [14]. This has been supported by a recent study that concluded that coil embolization could be cost-effective and minimally invasive in certain cases, depending on the morphology and anatomy of the lesion [15].

Our patient presented with abrupt hemorrhage needing resuscitation with volume expansion and blood products. Her source of bleeding was identified on the CT scan with a focus of active contrast extravasation interposed between the pancreatic head and the duodenum. Given her general improvement with volume expansion including both IVF and blood resuscitation, a trial of endovascular treatment with interventional radiology was deemed most appropriate. The benefits included avoidance of open procedure that would be technically difficult due to involvement of retroperitoneal structures and the size of the developing hematoma. Identification of a likely vessel source of bleeding on CT was deemed a good prognostic sign that endovascular treatment for bleeding would be safest. Although there is a lack of large-sized trials in management of visceral aneurysms associated with median arcuate ligament syndrome, there is a consensus from smaller trials that endovascular repair should be the first-line therapeutic management. As the safety and efficacy of endovascular techniques continue to grow, surgical procedures are more reserved for more severe and unstable cases. Surgical interventions can have difficulty accessing retroperitoneal structures; the treatment can range from resection to ligation of the aneurysms with or without arterial reconstruction and may need to be done at centers with experience and ability to complete pancreaticoduodenectomy [16]. In a recently published single-center review, Antoniak et al. support endovascular embolization pending ability to control bleeding, underlying comorbid conditions, and ability to determine one collateral pathway between the celiac artery and the SMA that are free from an aneurysm [16]. Our patient tolerated endovascular interventions but did require three angiograms to ultimately control her bleeding. She had initial improvement but with ongoing blood loss requiring reimaging. Due to her stability, repeat endovascular treatment was determined to be more advantageous as compared to surgical intervention for control of her bleeding. At each decision, surgical options were explored but the patient had no other comorbidities favoring surgery, and reattempt at endovascular treatment seemed promising. On the third mesenteric angiogram, aided by cone beam CT, successful identification of the bleeding source from a superior PDA branch supplying the aneurysm was identified and treated with coil embolization.

In this case, a multidisciplinary approach was effectively used to diagnose and treat a rare etiology of retroperitoneal hemorrhage utilizing conventional mesenteric angiography, cone beam CT with 3D reconstruction, and selective mesenteric transarterial embolization. The anatomical information gleaned from the procedure will also be utilized for future surgical consideration.
